# Semaglutide as a GLP-1 Agonist: A Breakthrough in Obesity Treatment

**DOI:** 10.3390/ph18030399

**Published:** 2025-03-12

**Authors:** Rui Salvador, Carla Guimarães Moutinho, Carla Sousa, Ana Ferreira Vinha, Márcia Carvalho, Carla Matos

**Affiliations:** 1 Faculty of Health Sciences, Fernando Pessoa University, Rua Carlos da Maia 296, 4200-150 Porto, Portugal; 29167@ufp.edu.pt (R.S.); carlamo@ufp.edu.pt (C.G.M.); sousasil@ufp.edu.pt (C.S.); acvinha@ufp.edu.pt (A.F.V.); 2 LAQV/REQUIMTE, Department of Chemical Sciences, Faculty of Pharmacy, University of Porto, R. Jorge de Viterbo Ferreira, 228, 4050-313 Porto, Portugal; 3 RISE-Health, Faculty of Health Sciences, Fernando Pessoa University, Fernando Pessoa Teaching and Culture Foundation, Rua Carlos da Maia 296, 4200-150 Porto, Portugal

**Keywords:** semaglutide, GLP-1 receptor agonist, obesity, weight loss therapy, metabolic disorders

## Abstract

This review addresses the role of semaglutide (SMG), a GLP-1 receptor agonist, in the treatment of obesity and its related comorbidities. Originally developed for type 2 diabetes (DM2), SMG has shown significant efficacy in weight reduction, with superior results compared to other treatments in the same class. Its effects include appetite suppression, increased satiety, and improvements in cardiovascular, renal, and metabolic parameters. Studies such as SUSTAIN, PIONEER, and STEP highlight its superiority compared to other GLP-1 receptor agonists and anti-obesity drugs. The oral formulation showed promising initial results, with higher doses (50 mg) showing weight losses comparable to those of subcutaneous administration. Despite its benefits, there are challenges, such as weight regain after cessation of treatment, gastrointestinal adverse effects, and variability of response. Future studies should explore strategies to mitigate these effects, identify predictive factors of efficacy, and expand therapeutic indications to other conditions related to obesity and insulin resistance. The constant innovation in this class of drugs reinforces the potential of SMG to transform treatment protocols for chronic weight-related diseases.

## 1. Introduction

Obesity is recognized as a chronic, complex, and multifactorial disease [1]. The World Health Organization (WHO) defines overweight and obesity as an abnormal or excessive accumulation of adipose tissue that poses a risk to health [2].

Adipose tissue plays a key role in regulating energy metabolism and body homeostasis [3]. However, excessive adiposity, especially in the waist area, translates into an increased risk of various chronic non-communicable diseases, namely cardiovascular disease, type 2 diabetes (DM2), hypertension, dyslipidemia, insulin resistance, sleep apnoea, hepatic steatosis, some forms of cancer, depression, and other comorbidities, ultimately reducing the quality of life of those who suffer from this disease [4,5].

According to the WHO, obesity has reached epidemic proportions, estimating that more than one billion adults worldwide will be obese by 2030 [6]. The prevalence of obesity has increased rapidly in recent years, doubling since 1980. More than a third of the world’s population is currently classified as overweight or obese [7,8], and more than two-thirds of adults in the United States are overweight, with 41.9% classified as obese and 9.2% as severe obese [9].

Studies indicate that individuals with obesity have a higher frequency of utilization of health services, undergo a higher number of surgical procedures, and require more pharmacological interventions compared to individuals with a normal Body Mass Index (BMI) [10,11]. Individuals with obesity incur 30% higher medical costs compared to those with a normal BMI. Beyond its impact on healthcare costs, obesity affects labor productivity, increases rates of absenteeism and presenteeism, and is associated with a lower quality of life [12,13].

Weight loss is widely recommended for individuals with obesity or overweight who have comorbidities such as diabetes, hypertension, or dyslipidemias [14]. The therapeutic goal for most individuals should be a weight loss of 5 to 10% of body weight over a period of 6 to 12 months [15].

International guidelines emphasize that an effective treatment strategy should be multidisciplinary, incorporating lifestyle modifications such as dietary changes and increased physical activity [16]. However, while lifestyle interventions can lead to short-term success, maintaining weight loss over time remains a significant challenge [17]. Weight regain is often attributed to the difficulty in maintaining adherence to lifestyle modifications, as well as the body’s physiological adaptation in response to weight loss [14].

For this reason, pharmacological therapy may be considered as a complement to lifestyle changes in individuals with a BMI ≥ 30 kg/m^2^ or BMI ≥ 27 kg/m^2^ in the presence of at least one associated comorbidity [18], who have difficulty adhering to treatment and are unable to achieve the required weight loss (>5% of initial weight) [19] after 6 months of lifestyle change interventions [19,20,21,22].

In recent years, interest in GLP-1 receptor agonists has surged, driven by their dual benefits in glycemic control and weight loss among individuals with DM2 and other metabolic conditions [23]. Several drugs within this class have received approval from the European Medicines Agency (EMA) for weight management, including orlistat, the naltrexone/bupropion combination (Mysimba^®^), liraglutide (Saxenda^®^), and tirzepatide (Mounjaro^®^). This study will focus on the effects of semaglutide (SMG) on weight loss in individuals with or without diabetes and other comorbidities.

### Literature Search

The literature search was conducted using the PubMed database. The initial search was performed without restrictions on publication date or language, using the terms ‘GLP-1’ (or ‘Glucagon-like peptide 1 agonist’) and ‘obesity’ (or ‘obese’ or ‘weight loss’ or ‘body weight’). Based on the results of this preliminary search, a more targeted search was carried out, focusing specifically on SMG to identify key studies—particularly randomized controlled trials or studies with an active comparison group—investigating its effects on body weight. To refine the search, filters related to study design were applied, including ‘Clinical Study’, ‘Clinical Trial’, ‘Clinical Trial, Phase I’, ‘Clinical Trial, Phase II’, ‘Clinical Trial, Phase III’, ‘Clinical Trial, Phase IV’, ‘Comparative Study’, ‘Controlled Clinical Trial’, ‘Multicenter Study’, and ‘Randomized Controlled Trial’. Only randomized controlled studies (or studies with an active comparison group) conducted in humans and evaluating SMG’s effects on weight loss were included.

In addition to the PubMed search, a complementary strategy was employed to identify potentially relevant studies that might have been missed. This included (i) screening the reference list of included studies to identify additional relevant publications; (ii) conducting a Google Scholar search to find more recent studies that cited the included studies; (iii) reviewing reference lists of systematic reviews on SMG to identify any overlooked studies; and (iv) performing targeted searches based on specific terms related to SMG trials (e.g., SUSTAIN or PIONEER).

The most relevant SMG studies were selected and analyzed according to the sample size per intervention group, presence of comorbidities, characteristics of the SMG, placebo or active comparator intervention (dosage, frequency, and duration), and significant weight loss outcomes. Additionally, a search was conducted on the ClinicalTrials.gov website to identify ongoing clinical trials investigating SMG.

## 2. GLP-1 Receptor Agonists

Initially developed for the treatment of DM2, GLP-1 receptor agonists not only improve glycemic control but also show significant effects on weight reduction. Their action includes delaying gastric emptying, suppressing appetite through hypothalamic signals, and increasing the feeling of satiety, which makes them promising for managing obesity in individuals with or without diabetes [24]. Drugs such as exenatide, liraglutide, dulaglutide, and SMG have revolutionized clinical practice by providing metabolic and cardiovascular benefits that go beyond glycemic control.

Exenatide was the first drug introduced in the GLP-1 receptor agonist class. It received approval from the U.S. Food and Drug Administration (FDA) in April 2005 as an adjunct therapy for glycemic control in patients with DM2 who were already taking metformin, a sulphonylurea, or a combination of both [25]. Early studies on twice-daily exenatide administration demonstrated its safety and efficacy, showing significant improvements in glycemic control and weight reduction in individuals with DM2 [26,27]. Exenatide later evolved into an extended-release formulation, yielding positive outcomes in individuals with DM2 [28,29]. Over time, its indications were expanded to include weight management in individuals with obesity or overweight without diabetes [30]. Long-term studies on once-weekly exenatide, with follow-up periods extending up to 6 years, confirmed its safety profile while demonstrating sustained glycemic control, moderate weight loss, and a low risk of hypoglycemia [31,32].

Liraglutide followed exenatide to be indicated and implemented for the treatment of DM2. It received FDA approval in January 2010 as an adjuvant therapy for glycemic control in patients with DM2. Even before its approval, a 2009 joint consensus statement from the American Diabetes Association and the European Association for the Study of Diabetes recommended the use of GLP-1 receptor agonists in patients where minimizing hypoglycemia or promoting weight loss was an important consideration [33]. Following the promising results of liraglutide on both glycemic control and weight loss in DM2, a higher-dose formulation (3.0 mg) was developed specifically for weight management. This formulation, marketed as Saxenda^®^, was approved for weight loss in individuals with obesity or overweight with associated comorbidities by the FDA in 2014 and by the EMA in 2015 [34,35,36,37].

Building on the success of exenatide and liraglutide, additional GLP-1 receptor agonists have since been developed, including dulaglutide [38], lixisenatide, SMG [39], and tirzepatide [40]. Among these, SMG has emerged as one of the most promising drugs due to its superior efficacy in both glycemic control and weight reduction compared to other GLP-1 receptor agonists [35,41,42,43]. Its advantages also include diversity in formulation (subcutaneous and oral), a range of dosing options, a lower risk of cardiovascular events [44], and extensive research supporting its benefits in conditions such as hepatic steatosis [45,46,47], sleep apnea [48,49], obesity-related heart failure with preserved ejection fraction [50,51,52], and DM2 prevention in prediabetic individuals [53,54].

SMG (Ozempic^®^) was approved by the FDA in December 2017 and by the EMA in February 2018 for the treatment of DM2. Following this, SMG was also approved for weight control in adults with obesity or overweight with comorbidities (Wegovy^®^) by the FDA in June 2021 and by the EMA in January 2022.

Proximal L-cells in the upper small intestine secrete GLP-1 in response to nutrient intake, which then activates GLP-1 receptors on chemosensory vagal afferents in the intestinal villi and hepatic portal vein, as well as on mechanosensory vagal afferents innervating the gut. Following large or nutrient-rich meals, GLP-1 levels may rise sufficiently to enter systemic circulation and directly influence the brain by acting on neuronal GLP-1 receptors in circumventricular organs, such as the area postrema [24].

**Figure 1:**
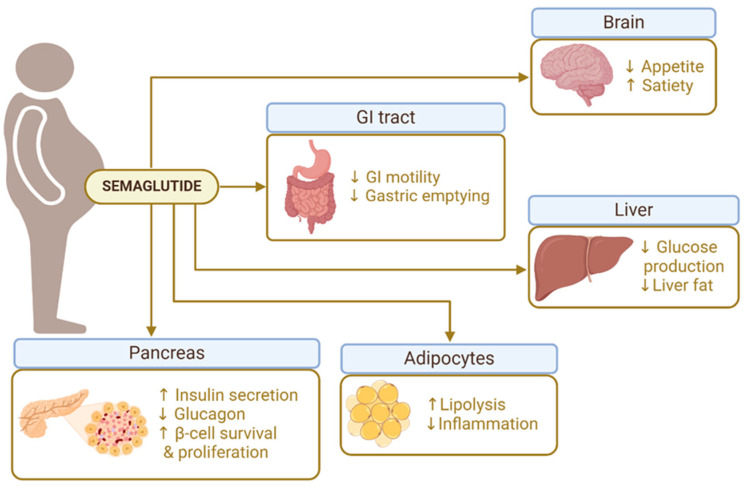
Mechanism of semaglutide for the management of obesity (Created in BioRender. Carvalho, M. (2025) https://BioRender.com/l84z782).

SMG weight-lowering effects are mediated through central and peripheral mechanisms that regulate appetite, energy balance, and metabolism (Figure 1). Its central effects are mediated by direct GLP-1 stimulation of anorexigenic neurons (proopiomelanocortin (POMC) and cocaine- and amphetamine-regulated transcript (CART) neurons) and inhibition of orexigenic neurons (neuropeptide Y (NPY) and agouti-related peptide (AgRP) neurons), located in the arcuate nucleus in the hypothalamus, reducing hunger and increasing satiety. This interaction is mediated by the hormone leptin and leads to decreased food intake, contributing to sustained weight loss [55,56]. Peripherally, SMG slows gastric emptying, prolonging digestion and promoting early satiety. This mechanism not only lowers calorie intake but also reduces postprandial glucose spikes, contributing to improved metabolic control [25]. Additionally, some studies suggest that GLP-1 receptor agonists may increase energy expenditure, possibly by enhancing thermogenesis in brown adipose tissue [57].

SMG has structural modifications that give it a half-life of approximately one week. This is achieved by substituting amino acids and attaching a fatty acid to its peptide chain, allowing for greater affinity for albumin and protection against degradation by dipeptidyl peptidase-4 (DPP-4) [39,58]. As a result, subcutaneous SMG is administered by weekly subcutaneous injection at doses of 0.25 mg, 0.5 mg, 1.0 mg, 1.7 mg, and 2.4 mg. Alternatively, it may be administered daily orally (Ribelsus^®^) in doses of 3 mg, 7 mg, or 14 mg. This GLP-1 analog has 94% amino acid sequence homology and mimics the physiological effects of endogenous GLP-1, providing benefits in both glycemic control and body weight regulation [39].

Considering the currently marketed GLP-1 receptor agonists, differences in binding affinity, receptor activation dynamics, and secondary effects can lead to variations in clinical outcomes [59,60]. Short-acting molecules, such as exenatide and lixisenatide, bind to the GLP-1 receptor transiently, closely mimicking the natural pulsatile secretion of endogenous GLP-1. Due to their shorter half-life, they typically require twice-daily or daily dosing and have relatively modest effects on fasting glucose levels and weight loss. In contrast, long-acting molecules, such as liraglutide, SMG, dulaglutide, and tirzepatide, provide continuous receptor activation, resulting in prolonged appetite suppression and insulinotropic effects. This sustained action enhances glycemic control and promotes greater weight loss, making these agents more effective for long-term weight management. Among these, weekly administration of SMG at a 2.4 mg dose showed a greater average weight loss compared to liraglutide, the other GLP-1 receptor agonist approved specifically for weight management [59,60].

## 3. Main Studies and Results of Semaglutide for Weight Loss

The early clinical trials of SMG (phase 2 and phase 3a) were designed to determine the optimal dose of subcutaneous SMG compared to placebo or liraglutide [59] and to assess the safety and efficacy of both subcutaneous and oral SMG compared to placebo [60] in individuals with DM2. The phase 2 trial demonstrated a clear dose-dependent effect of SMG, with no unexpected safety or tolerability concerns, establishing for phase 3 studies that the optimal weekly subcutaneous doses of SMG should be 0.5 and 1.0 mg, with dose escalation every 4 weeks [59]. The phase 3a trial showed that while adverse events were more frequent with SMG compared to sitagliptin, its overall safety profile was similar to that of other GLP-1 receptor agonists [60]. Both studies showed a statistically significant reduction in body weight with SMG compared to placebo or liraglutide (Table 1), further supporting its efficacy as a weight-loss intervention.

The most relevant clinical studies on SMG are grouped by the main research programs that established its efficacy and safety for both subcutaneous (Ozempic^®^) and oral (Rybelsus^®^) administration in the treatment of DM2. Over time, the approved indications for subcutaneous SMG (Wegovy^®^) were expanded to include weight management in individuals with obesity or excess weight associated with comorbidities such as diabetes, hypertension, dyslipidemia, obstructive sleep apnea, cardiovascular disease, or osteoarthritis of the knee, where reducing fat mass plays a crucial role in both treatment and secondary prevention.

Other groups of subsequent studies have investigated the effects of SMG on renal and cardiovascular systems while also exploring its potential benefits in broader patient populations that may experience improved glycemic control and significant weight loss.

### 3.1. Semaglutide Unabated Sustainability in Treatment of Type 2 Diabetes (SUSTAIN)

The first group of clinical studies was Semaglutide Unabated Sustainability in Treatment of Type 2 Diabetes (SUSTAIN), which evaluated the weekly subcutaneous administration of SMG in individuals with DM2 [61,62,63]. The SUSTAIN 1 study [64] demonstrated the efficacy of SMG (doses of 0.5 mg and 1 mg) compared to placebo in improving glycated hemoglobin (HbA1c) in patients with DM2. The starting dose was 0.25 mg per week, with the dose doubled every 4 weeks until it reached 0.5 to 1.0 mg per week.

The SUSTAIN studies were designed to evaluate the efficacy and safety of SMG compared to placebo or other drugs (including other GLP-1 receptor agonist drugs) in patients with DM2 [61]. In fact, these studies showed that, in addition to SMG being effective in reducing and controlling HbA1c, it also had a statistically significant effect on weight loss (Table 2). Higher doses (1.0 mg vs. 0.5 mg) led to greater weight loss [64,65,66,67,68]. Weight loss was independent of race/ethnicity [62], age group, or initial BMI [69], but discontinuation of the SMG intervention was higher in elderly individuals [70]. A relevant proportion (15–27%) of individuals experienced nausea or vomiting after taking SMG, but this adverse effect had a minor contribution to weight loss [69]. In addition to weight loss, overall satisfaction after taking SMG was reported to be higher when compared to other medications or placebo [63].

When SMG was compared to other GLP-1 receptor agonist medications (exenatide extended-release, dulaglutide, and liraglutide), SMG showed greater weight loss [71,72,73]. Based on simulation models, it is estimated that replacing liraglutide, dulaglutide, or exenatide extended-release treatment with SMG (Ozempic^®^) results in additional reductions in body weight of between 2% and 4% [74].

SUSTAIN 6 [67] demonstrated the safety of SMG in relation to cardiovascular events and other relevant long-term outcomes in patients with DM2 who were at high cardiovascular risk. The SUSTAIN study group was instrumental in obtaining approval of SMG (Ozempic^®^) for the treatment of DM2.

The SUSTAIN trials demonstrated the non-inferiority of SMG as treatment for DM2, consistently reducing HbA1c levels and body weight with superior results compared to other standard therapies and significant cardiovascular benefits (SUSTAIN 6). Key strengths include well-designed, randomized controlled studies and broad comparisons with existing treatments. Despite the relatively short duration of some trials limiting long-term safety assessments, the SUSTAIN trials confirmed SMG as a promising therapeutic option, warranting further long-term studies to fully assess its safety and cardiovascular impact.

**Table 2:** Results of the main randomized controlled trials of subcutaneous semaglutide (SMG) in the SUSTAIN studies in patients with type 2 diabetes.

Reference	Study Design	Population	Intervention	Results in Body Weight
[64] [SUSTAIN 1]	Placebo-controlled, double-blind trial	n = 242	SMG Initial dose 0.25 mg, with a double dose every 4 weeks up to 0.5 to 1.0 mg/week (sc, 30 weeks)	- ↑↑ reduction for SMG (−3.7 and −4.5 kg) vs. placebo (−1.0 kg). - % patients attaining BW reduction ≥5% higher for SMG (37% and 45%) vs. 7% after placebo.
		n = 129	Placebo Weekly dose (sc, 30 weeks)	
[65] [SUSTAIN 2]	Double-blind, double-dummy, active-controlled trial	n = 818	SMG Initial dose 0.25 mg, with a double dose every 4 weeks up to 0.5 to 1.0 mg/week (sc, 56 weeks) Sitagliptine 100 mg/day (oral, 56 weeks)	- ↑↑ reduction for SMG (−4.3 and −6.1 kg) vs. sitagliptin (−1.9 kg). - % patients achieving BW reduction ≥ 5% higher for SMG (46% and 62%) vs. 18% after sitagliptin.
		n = 407		
[71] [SUSTAIN 3]	Open-label, parallel-group trial	n = 404	SMG Initial dose 0.25 mg, with a doubling dose every 4 weeks up to 1.0 mg/week (sc, 56 weeks)	- ↑↑ reduction for SMG (−5.6 kg) vs. exenatide (−1.9 kg). - % patients reaching BW reduction ≥ 5% higher for SMG (52%) vs. 17% after exenatide.
		n = 405	Exenatide (Extended release) 2 mg/ week (sc, 56 weeks)	
[66] [SUSTAIN 4]	Open-label, parallel-group trial	n = 618	SMG Initial dose 0.25 mg, with a double dose every 4 weeks up to 0.5 to 1.0 mg/week (sc, 30 weeks)	- ↑↑ reduction for SMG (−3.5 and −5.2 kg) vs. insulin glargine (+1.2 kg). - % patients achieving BW reduction ≥ 5% higher for SMG (37% and 51%) vs. 5% after insulin glargine.
		n = 324	Insulina glargina (IGlar) 10 IU/ day (sc, 30 weeks)	
[68] [SUSTAIN 5]	Placebo-controlled, double-blind trial	n = 263	SMG Initial dose 0.25 mg, with a doubling dose every 4 weeks up to 0.5 to 1.0 mg/ week (sc) as an add-on to basal insulin (30 weeks) Placebo Dose equivalent (sc) as an add-on to basal insulin (30 weeks)	- ↑↑ reduction for SMG (−3.7 and −6.4 kg) vs. placebo (−1.4 kg). - % patients attaining BW reduction ≥5% higher for SMG (42% and 66%) vs. 11% after placebo.
		n = 133		
[67] [SUSTAIN 6]	Placebo-controlled, double blind trial	n = 1648	SMG Starting dose 0.25 mg with a doubling dose every 4 weeks up to 0.5 to 1.0 mg/week (ss, 104 weeks) Placebo Initial dose 0.25 mg, with a doubling dose every 4 weeks up to 0.5 to 1.0 mg/ week (sc, 104 weeks)	- ↑↑ reduction for SMG (−3.6 and −4.9 kg) vs. placebo (−0.7 and 0.5 kg).
		n = 1649		
[73] [SUSTAIN 7]	Open-label, parallel-group trial	n = 601	SMG Initial dose 0.25 mg, with a doubling dose every 4 weeks up to 0.5 to 1.0 mg/week (sc, 40 weeks) Dulaglutide 0.75 to 1.5 mg/week (sc, 40 weeks)	- ↑ reduction for SMG 0.5 and 1.0 mg vs. dulaglutide 0.75 and 1.5 mg (−0.4 and −0.4 kg) - % patients reaching BW reduction ≥ 5% higher for SMG 0.5 to 1.0 mg (44% and 63%) vs. 23% and 30% of those with dulaglutide 0.75 to 1.5 mg.
		n = 598		
[75] [SUSTAIN 8]	Double-blind, parallel-group trial	n = 367	SMG Progressive dose up to 1.0 mg/week (sc, 52 weeks)	- ↑↑ reduction for SMG (−5.3 kg) vs. canagliflozin (−4.2 kg). - % patients achieving BW reduction ≥ 15% (super-responders) for SMG was 7%.
		n = 372	Canagliflozin Progressive dose up to 300 mg/week (oral, 52 weeks)	
[76] [SUSTAIN 9]	Placebo-controlled, double-blind trial	n = 147	SMG Initial dose 0.25 mg, with a double dose every 4 weeks up to 1.0 mg/ week (sc, 30 weeks)	- ↑↑ reduction for SMG (−4.7 kg) vs. placebo (−0.9 kg). - % patients reaching BW reduction ≥ 5% higher for SMG (50%) vs. 8% after placebo.
		n = 147	Placebo Initial placebo dose 0.25 mg, with a doubling dose every 4 weeks up to 1.0 mg/week (sc, 30 weeks)	
[72] [SUSTAIN 10]	Open-label, active-controlled, parallel-group trial	n = 287	SMG Initial dose 0.25 mg, with a double dose every 4 weeks up to 1.0 mg/week (sc, 30 weeks)	- ↑↑ reduction for SMG (−5.8 kg) vs. liraglutide (−1.9 kg). - % patients attaining BW reduction ≥ 5% higher for SMG (56%) vs. 18% after liraglutide.
		n = 282	Liraglutide Progressive dose up to 1.2 mg/week achieved in 1 to 2 weeks (sc, 30 weeks)	
[77] [SUSTAIN 11]	Open-label, active-controlled, parallel-group trial	n = 806	SMG 1.0 mg/week (sc, 52 weeks) in addition to metformin (1500–3000 mg) Aspartic insulin 3 times/day (sc) up to a total of 100 U/mL/week (52) weeks in addition to metformin (1500–3000 mg)	- ↑↑ reduction for SMG (−4.1 kg) vs. insulin aspartic (+2.8 kg).
		n = 831		

Abbreviations: BW—body weight, sc—subcutaneous, SMG—semaglutide, ↑—higher, ↑↑—significantly higher.

### 3.2. Peptide InnOvatioN for Early diabEtes tReatment (PIONEER)

The second group of clinical studies was Peptide InnOvatioN for Early diabEtes tReatment (PIONEER), which investigated oral SMG (Rybelsus^®^) for the treatment of DM2. Rybelsus^®^ (3 mg, 7 mg or 14 mg per day) was the first oral GLP-1 receptor agonist. The oral SMG was usually started at a dose of 3 mg, then increased to 7 mg in 4 weeks and 14 mg in 8 weeks. The use of dose progression was aimed at improving gastrointestinal tolerability, since initial studies found gastrointestinal adverse effects at high doses [60]. Rybelsus^®^ was taken in the morning on an empty stomach with up to half a glass of water (approximately 120 mL) 30 min before any other food, drink, or other oral medication, since the absorption of oral SMG is affected by food and fluids in the stomach.

This group of PIONEER studies was important in demonstrating that oral SMG is effective for controlling glycemia and reducing weight in patients with DM2 [68,76,78,79,80,81,82,83] and as a non-inferior option compared to injectables for glycemic control [84,85,86].

The PIONEER 6 study [87] investigated the effect of oral SMG in individuals with DM2 and cardiovascular or chronic kidney disease. This study focused on cardiovascular outcomes and showed that oral SMG was associated with a reduced risk of major adverse cardiovascular events.

The PIONEER 7 study [79] carried out a cross-over extension to assess the efficacy of switching from sitagliptin medication to oral SMG in patients with DM2. In short, the first part of PIONEER 7 lasted 52 weeks, where participants were randomized to take SMG (up to 3 mg, 7 mg, or 14 mg per day) or sitagliptin (100 mg per day). Both groups continued with their previous glucose control medication. In the second part of the study [88], the effect of switching from sitagliptin to oral SMG was evaluated over a further 52 weeks of study. Participants in the sitagliptin group were randomized to receive oral SMG or to continue with sitagliptin (following the same protocol and dosage as in the previous study). The study showed that switching from sitagliptin to oral SMG maintained HbA1c reductions, helped more patients achieve HbA1c targets with less use of additional glucose-lowering medications, and resulted in additional body weight reductions.

The PIONEER studies were fundamental to the approval of oral SMG (Rybelsus^®^) for the treatment of DM2. Oral SMG provided consistent reductions in HbA1c and body weight across diverse patient populations. Additionally, the favorable cardiovascular safety profile (PIONEER 6) supports its long-term use. However, its efficacy is slightly lower compared to the subcutaneous formulation, and strict adherence is required for optimal absorption, as it must be taken on an empty stomach with minimal water. Gastrointestinal side effects, including nausea and vomiting, were common, potentially affecting tolerability. Despite these limitations, oral SMG represents a major advancement in diabetes treatment, warranting further studies to assess long-term safety and adherence in real-world settings.

The PIONEER group of studies showed that oral SMG was effective in reducing body weight with a dose-dependent effect, where higher doses had a greater effect on weight loss (Table 3). More recently, an extension of the PIONEER studies was published entitled PIONEER PLUS [89], which compared once-daily oral SMG 14 mg, 25 mg, or 50 mg for 68 weeks. The higher dose oral SMG (25 mg and 50 mg) was superior to the 14 mg dose in reducing body weight. Gastrointestinal disturbances, which were mostly mild to moderate, occurred more frequently with oral SMG 25 mg and 50 mg than with the 14 mg dose, but without raising concerns about potential safety risks.

The PIONEER REAL initiative was launched to assess real-world clinical outcomes of oral SMG. This pooled analysis included seven noninterventional, multicenter, phase 4 studies (34–44 weeks) evaluating its use in adults with DM2 in routine practice. Data from 1615 participants across seven countries who had not previously used injectable glucose-lowering therapy showed significant reductions in HbA1C and body weight regardless of age, DM2 duration, or dose. These benefits were consistent across clinical settings and accompanied by improved treatment satisfaction. This analysis complements the PIONEER clinical program and provides valuable insights into the real-world use and safety of oral SMG [90].

**Table 3:** Results of the main randomized controlled trials of oral semaglutide in the PIONEER studies in patients with type 2 diabetes.

Reference	Study Design	Population	Intervention	Results in Body Weight
[78] [PIONEER 1]	Placebo-controlled, double-blind trial	n = 525	SMG Progressive dose from 3 mg (increases every 4 weeks) up to 3 mg, 7 mg, or 14 mg/day (oral), 26 weeks	- **↑↑** reduction for SMG (−1.5 to −3.7 kg) vs. placebo (−1.4 kg). - % patients attaining BW loss ≥ 5% greater for SMG (20–41%) vs. 15% after placebo.
		n = 178	Placebo Daily dose (oral), 26 weeks	
[91] [PIONEER 2]	Open-label trial	n = 400	SMG Progressive dose (increases from 3 mg, to 7 mg at 4 weeks, and 14 mg at 8 weeks) up to 14 mg daily (oral), 52 weeks	- **↑↑** loss for SMG (−4.7 kg) vs. empagliflozin (−3.8 kg). - % patients reaching BW loss ≥ 5% similar for SMG (47%) vs. 42% after empagliflozin.
		n = 387	Empagliflozin Starting dose of 10 mg/ day and increased to 25 mg/day at 8 weeks (oral), 52 weeks	
[82] [PIONEER 3]	Double-blind trial	n = 1396	SMG Progressive dose from 3 mg (increases every 4 weeks) up to 3 mg, 7 mg, or 14 mg daily (oral), 78 weeks, in addition to metformin and in half of sulfonylurea cases	- **↑↑** drop for SMG vs. sitagliptin (−0.8 kg for 3 mg, −1.7 kg for 7 mg, and −2.1 kg for 14 mg). - % patients accomplishing BW reduction ≥ 5% superior for SMG (23–36%) vs. 15% after sitagliptin.
		n = 467	Sitagliptin 100 mg daily (oral), 78 weeks, in addition to metformin and in half of sulfonylurea cases	
[84] [PIONEER 4]	Placebo-controlled, double-blind trial	n = 241	SMG Progressive dose (increases from 3 mg, to 7 mg at 4 weeks, and 14 mg at 8 weeks) up to 14 mg daily (oral), 52 weeks, in addition to metformin (≥1500 mg)	- **↑↑** decrease for SMG (−5.0 kg) vs. liraglutide (−3.1 kg) and placebo (−1.2 kg). - % patients reaching BW loss ≥5% significantly greater for SMG (49%) vs. 26% after liraglutide and 12% after placebo.
		n = 248	Liraglutide Progressive dose of 0.6 mg daily up to 1.2 mg daily after one week and 1.6 mg at two weeks (sc), 52 weeks, in addition to metformin (≥1500 mg)	
		n = 125	Placebo Equivalent doses of SMG (oral) and liraglutide (sc), 52 weeks, in addition to metformin (≥1500 mg)	
[81] [PIONEER 5]	Placebo-controlled, double-blind trial	n = 133	SMG Progressive dose (increases from 3 mg, to 7 mg at 4 weeks, and 14 mg at 8 weeks) up to 14 mg daily (oral), 26 weeks, in addition to metformin or sulfonylurea	- **↑↑** decline for SMG (−3.7 kg) vs. placebo (−1.1 kg). - % patients attaining BW reduction ≥ 5% larger for SMG (36%) vs. 10% after placebo.
		n = 141	Placebo Equivalent doses (oral), 26 weeks, in addition to metformin or sulfonylurea	
[87] [PIONEER 6]	Placebo-controlled, double-blind trial	n = 1347 (DM2 and chronic cardiovascular or CKD)	SMG Progressive dose (increases from 3 mg, to 7 mg at 4 weeks, and 14 mg at 8 weeks) up to 14 mg daily (oral), 69 weeks	- **↑↑** BW reduction for SMG (−4.2 kg) vs. placebo (−0.8 kg).
		n = 1435 (DM2 and chronic cardiovascular or CKD)	Placebo Equivalent doses (oral), 69 weeks	
[79] [PIONEER 7]	Open-label trial	n = 211	SMG Progressive dose starting at 3 mg daily (oral) and progressing to 8 weeks based on HbA1c levels (up to 3 mg, 7 mg, or 14 mg), 52 weeks, in addition to prior medication to control glucose	- **↑↑** BW reduction for SMG (−2.9 kg) vs. sitagliptin (−0.8 kg).
		n = 228	Sitagliptin 100 mg daily (oral), 52 weeks, in addition to metformin and in half of sulfonylurea cases, in addition to prior medication to control glucose	
[88] [PIONEER 7]	Open-label trial (cross-over)	n = 100	SMG /Sitagliptin 52-week follow-up: 198 sitagliptin patients randomized to continue or switch to SMG (same protocol/dosage)	- **↑↑** BW decrease for patients switched to SMG (−2.9 kg) vs. those continuing sitagliptin (−1.0 kg).
		n = 98		
[92] [PIONEER 8]	Placebo-controlled, double-blind trial	n = 546	SMG Doses of 3 mg, 7 mg, and 14 mg (with progressive doses up to 7 mg at week 4 and 14 mg at week 8)/day (oral), 52 weeks, with or without added metformin	- **↑↑** progressive, dose-dependent loss for SMG (−1.0 to −4.1 kg) vs. placebo (+0.6 kg). - % patients attaining BW drop ≥ 5% dose-dependent (25%, 36%, and 49% with SMG) vs. 5% after placebo.
		n = 184	Placebo Equivalent doses (oral), 52 weeks, with or without added metformin	
[86] [PIONEER 9]	Placebo-controlled, double blind trial: SMG and placebo; open-label trial: liraglutide	n = 146	SMG Doses of 3 mg, 7 mg, and 14 mg (with progressive doses up to 7 mg at week 4 and 14 mg at week 8)/day (oral), 52 weeks	- **↑↑** reduction for SMG (dose-dependent −1.1 to −1.8 kg) vs. placebo (−0.4 kg), but not statistically different vs. liraglutide (−1.4 kg). - % patients reaching BW decrease ≥ 5% greater for SMG (38% for 7 mg, 12% for 14 mg) vs. 7% after liraglutide and 8% after placebo. Only 4% for 3 mg of SMG.
		n = 48	Liraglutide Progressive dose of 0.3 mg/day up to 0.9 mg/day at two weeks (sc), 52 weeks	
		n = 49	Placebo SMG-equivalent doses (oral), 52 weeks	
[85] [PIONEER 10]	Open-label, active-controlled trial	n = 362	SMG Doses of 3 mg, 7 mg, and 14 mg (with progressive doses up to 7 mg at week 4 and 14 mg at week 8)/day (oral), 52 weeks	- **↑↑** loss for SMG (−1.0 kg for 7 mg, −1.9 kg for 14 mg) vs. dulaglutide (+1.1 kg); 3 mg of SMG had no clinical impact (+0.1 kg). - % patients attaining BW reduction ≥ 5% superior for SMG (17% for 7 mg, 25% for 14 mg) vs. 7% after dulaglutide. Only 5% for 3 mg of SMG.
		n = 61	Dulaglutide 0.75 mg/week (sc), 52 weeks	
[83] [PIONEER 11]	Placebo-controlled, double-blind trial	n = 361	SMG Doses of 3 mg, 7 mg, and 14 mg (with progressive doses up to 7 mg at week 4 and 14 mg at week 8)/day (oral), 26 weeks	- **↑↑** decline for SMG (−2.6 kg for 7 mg, −3.6 kg for 14 mg) vs. placebo (−1.0 kg); 3 mg of SMG was similar to placebo (−1.3 kg) - % patients attaining BW reduction ≥ 5% dose-dependent (15%, 24%, and 36% with SMG) vs. 9% after placebo.
		n = 121	Placebo Equivalent doses (oral), 26 weeks	
[80] [PIONEER 12]	Double-blind, double-dummy- active-controlled, parallel-group trial	n = 1082	SMG Doses of 3 mg, 7 mg, and 14 mg (with progressive doses up to 7 mg at week 4 and 14 mg at week 8)/day (oral), 26 weeks, and in some cases with continued metformin	- **↑↑** reduction for SMG vs. sitagliptin (−0.9 kg for 3 mg, −2.2 kg for 7 mg, and -3.0 kg for 14 mg). - % patients reaching BW decrease ≥5% larger for SMG (dose-dependent 16–45%) vs. 8% after sitagliptin.
		n = 359	Sitagliptin 100 mg daily (oral), 26 weeks, and in some cases with continued metformin	

Abbreviations: BW—body weight, CKD—chronic kidney disease, sc—subcutaneous, ↑↑—significantly higher.

### 3.3. Semaglutide Treatment Effect in People with Obesity (STEP)

Following the good results of the SUSTAIN studies (subcutaneous SMG) in controlling HbA1c and especially with subsequent weight loss, the need arose to expand the indications for SMG beyond the treatment of DM2. In this context, the Semaglutide Treatment Effect in People with Obesity (STEP) study group was set up with the aim of expanding the indications for SMG by investigating the effect of a new dose of 2.4 mg SMG (Wegovy^®^) in individuals with obesity or excess weight associated with comorbidities.

In the STEP studies, SMG (2.4 mg per week, Wegovy^®^) was administered in conjunction with intensive behavioral therapy (sessions every 4 weeks) aimed at weight loss. SMG was administered weekly by subcutaneous injection at an initial dose of 0.25 mg, which was doubled every 4 weeks until the desired dose of 1 mg or 2.4 mg per week was reached. The subcutaneous dose applied in the STEP studies (2.4 mg) is higher than that administered in the SUSTAIN studies (0.5 mg to 1.0 mg).

The STEP 1, STEP 3–6, and STEP 8 studies enrolled participants with obesity or overweight associated with comorbidities (mostly hypertension, dyslipidemia, obstructive sleep apnea, or cardiovascular disease) and demonstrated the superiority of SMG over placebo in weight loss [53,93,94,95,96,97,98]. In particular, the STEP 1 study, which originally lasted 68 weeks, was extended for an additional 52 weeks, during which the drug (SMG or placebo) was withdrawn and the washout effect of the drug was investigated [99]. While the participants in the placebo group regained all their weight, the participants in the SMG group gained +12.0 kg at 120 weeks (compared to 68 weeks when they had lost −18.1 kg), but the final result was still a weight loss of −6.1 kg. The STEP 4 study [93] had a cross-over design, where at 20 weeks a new randomization took place where participants from the original SMG group were randomly selected (in a 2:1 ratio) to continue with SMG or receive placebo and followed for a further 48 weeks. The SMG group initially lost −11.2 kg after the first 20 weeks, and after randomization, the participants who continued with SMG lost a further −7.1 kg, while those who switched to placebo gained +6.1 kg. The STEP 8 study showed that in addition to being superior to placebo, SMG was also superior to daily administration of liraglutide for weight loss [94]. The STEP 5 study [100] showed that in addition to weight loss, SMG was effective in controlling satiety by reducing food cravings.

In particular, the STEP 2 study [101] evaluated the effects of SMG (compared to placebo) on body weight in individuals with DM2 and obesity or overweight, STEP 7 [102] in individuals with obesity or overweight and at least one comorbidity (including DM2 or no DM2), and STEP 10 [53] in individuals with obesity and prediabetes. All three studies showed that SMG was superior to placebo in reducing body weight (Table 4).

A subsequent combined analysis of the STEP 1–4 studies showed that gastrointestinal adverse events (nausea, vomiting, diarrhea, and constipation) were more common with SMG than with placebo but typically mild to moderate and transient, with weight loss being largely independent of gastrointestinal adverse events [103]. In addition, a further analysis of the STEP 1–4 studies showed that weight loss was associated with an improvement in health-related quality of life [104].

The STEP group also carried out two other studies in subpopulations with obesity. The STEP 9 study investigated the effect of SMG (Wegovy^®^) in individuals with obesity and with knee osteoarthritis, where weight loss plays a crucial role [105]. The research demonstrated that the percentage reduction in body weight was significantly higher with SMG compared to placebo [106]. The STEP TEENS study [107] evaluated the effect of SMG (versus placebo) in adolescents with obesity. The findings indicated that the proportion of adolescents who reached normal weight or who dropped from obesity to overweight were higher among those who received SMG, also resulting in an improvement of at least one BMI category in almost three out of four adolescents who received SMG.

This study was important in the context of the high prevalence of obesity in the adolescent population [37,108] and the relevance of implementing effective early treatment in children, which demonstrates an increased risk of continued obesity into adolescence and adulthood [109,110,111]. Although first-line recommendations for the treatment of obesity in children and adolescents involve multifactorial lifestyle modifications (diet and increased physical activity) and behavioral change components that aim to sustain these modifications [112,113,114,115], these have been shown to be ineffective in the long term for a clinically meaningful and lasting reduction in body weight and improvement in BMI [114,116,117]. In this context, SMG can play a relevant role in aiding weight loss associated with conventional interventions.

In summary, the STEP studies were important to confirm the efficacy of SMG in weight loss in individuals with obesity or overweight associated with comorbidities (Table 4), leading to the approval of the indication of SMG 2.4 mg (Wegovy^®^) for weight loss in this subpopulation. Key strengths include well-designed randomized controlled trials demonstrating significant weight loss and metabolic benefits. However, major limitations include weight regain after treatment discontinuation, highlighting the need for long-term therapy. Gastrointestinal side effects such as nausea and vomiting were common and may affect adherence. In addition, weight loss was less pronounced in people with DM2, and long-term cardiovascular safety data in obese populations remain limited. Despite these challenges, SMG 2.4 mg represents a breakthrough in the treatment of obesity and warrants further study of its long-term effects.

**Table 4:** Results of the main randomized controlled trials of subcutaneous semaglutide in the STEP trials in individuals with obese or overweight associated with comorbidities.

Reference	Study Design	Population	Intervention	Results in Body Weight
[95] [STEP 1]	Placebo-controlled, double-blind trial	n = 1059 *	SMG 2.4 mg/week, 68 weeks	- **↑↑** loss for SMG (−4.7 kg) vs. placebo (−0.9 kg). - % patients attaining BW reduction ≥ 5% significantly superior for SMG (92%) vs. 33% after placebo.
		n = 499 *	Placebo Equivalent doses, 68 weeks	
[99] [STEP 1 extension]	Placebo-controlled, double-blind (cross-over) trial	n = 197 * n = 57 *	SMG and placebo Follow-up of the previous study for an additional 52 weeks with intervention washout	- Subgroups with ↑↑ BW losses from week 0 → 68 tended to regain BW from week 68 → 120, but still maintained greater overall BW loss from week 0 → 120. - BW in SMG group: week 0 → 68: −18.1 kg; week 68 → 120: +12.0 kg; week 0 → 120: −6.1 kg; BW in placebo group: week 0 → 68: -2.2 kg; week 68 → 120: +2.0 kg; week 0 → 120: 0 kg (regained full BW).
[101] [STEP 2]	Placebo-controlled, double-blind trial	n = 781, and with DM2	SMG Initial dose of SMG 0.25 mg, with a doubling dose every 4 weeks up to 1 mg or 2.4 mg/week, 68 weeks	- **↑↑** decrease for SMG (−6.9 to −9.7 kg) vs. placebo (−3.5 kg). - % patients reaching BW loss ≥ 5% greater for SMG (dose-dependent 59% and 73%) vs. 28% after placebo.
		n = 383, and with DM2	Placebo Equivalent doses, 68 weeks	
[118] [STEP 3]	Placebo-controlled, double-blind trial	n = 407 *	SMG Initial dose of SMG 0.25 mg, with a doubling dose every 4 weeks up to 2.4 mg/week, 68 weeks, combined with intensive behavioral therapy (30 sessions)	- **↑↑** reduction for SMG (−16.8 kg) vs. placebo (−6.2 kg). - % patients attaining BW loss ≥ 5% significantly larger for SMG (87%) vs. 6% after placebo.
		n = 204 *	Placebo Equivalent doses, 68 weeks, combined with intensive behavioral therapy (30 sessions)	
[93] [STEP 4]	Placebo-controlled, double-blind (cross-over) trial	n = 407, at least one comorbidity *	SMG All patients received an initial dose of 0.25 mg SMG, increasing every 4 weeks to 2.4 mg/week over 20 weeks. At 20 weeks, they were randomized (2:1) to continue SMG or receive placebo, with follow-up for another 48 weeks	- **↑↑** loss at 20 weeks with SMG (−11.2 kg); at 68 weeks, SMG group lost another −7.1 kg, while those switched to placebo gained +6.1 kg. - % patients reaching BW decrease ≥ 5% higher for SMG (89%) vs. 48% for those switched to placebo at 20 weeks.
		n = 204, at least one comorbidity *	Placebo Equivalent doses, from week 20 to 68	
[96] [STEP 5]	Placebo-controlled, double-blind (cross-over) trial	n = 148, at least one comorbidity *	SMG Initial dose of SMG 0.25 mg, with a doubling dose every 4 weeks up to 2.4 mg/week, 104 weeks	- **↑↑** decline for SMG (−16.1 kg) vs. placebo (−3.2 kg). - **↑** % patients attaining BW loss ≥5% greater for SMG (77%) vs. 34% after placebo.
		n = 134, at least one comorbidity *	Placebo Equivalent doses, 104 weeks	
[97] [STEP 6]	Placebo-controlled, double-blind trial	n = 291, at least one comorbidity *	SMG Starting dose of SMG 0.25 mg, with a doubling dose every 4 weeks up to 1.7 mg or 2.4 mg/week, 68 weeks	- **↑↑** reduction for SMG (−9.6% to −13.2%) vs. placebo (−2.1%). - % patients reaching BW decrease ≥ 5% superior for SMG (72% and 83%) vs. 21% after placebo.
		n = 100, at least one comorbidity *	Placebo Equivalent doses, 68 weeks	
[102] [STEP 7]	Placebo-controlled, double-blind trial	n = 291, at least one comorbidity * (with or without DM2 ^†^)	SMG Initial dose of SMG 0.25 mg, with a doubling dose every 4 weeks up to 2.4 mg/week, 44 weeks	- **↑↑** decrease for SMG (−12.1%) vs. placebo (−3.6%). - % patients attaining BW reduction ≥ 5% larger for SMG (77%) vs. 34% after placebo.
		n = 100, at least one comorbidity * (with or without DM2 ^†^)	Placebo Equivalent doses, 44 weeks	
[94] [STEP 8]	Placebo-controlled, double-blind trial	n = 126, at least one comorbidity *	SMG Initial dose of SMG 0.25 mg, with a double dose every 4 weeks up to 2.4 mg/week, 68 weeks	- **↑↑** reduction for SMG (−15.3 kg) vs. liraglutide (−6.4 kg) and placebo (−1.4 kg). - % patients attaining BW loss ≥ 5% higher for SMG (87%) vs. 58% after liraglutide and 30% after placebo.
		n = 127, at least one comorbidity *	Liraglutide Initial daily dose of 0.6 mg, with progressive dose up to 3.0 mg achieved in four weeks, 68 weeks	
		n = 125, at least one comorbidity *	Placebo Equivalent doses, 68 weeks	
[106] [STEP 9]	Placebo-controlled, double-blind trial	n = 271, and with osteoarthritis of the knee	SMG Initial dose of SMG 0.25 mg, with a double dose every 4 weeks up to 2.4 mg/week, 68 weeks	- **↑↑** BW decrease for SMG (−13.7%) vs. placebo (−3.2%).
		n = 136, and with osteoarthritis of the knee	Placebo Equivalent doses, 68 weeks	
[53] [STEP 10]	Placebo-controlled, double-blind trial	n = 129, and with prediabetes	SMG Initial dose of SMG 0.25 mg, with a doubling dose every 4 weeks up to 2.4 mg/week, 52 weeks	- **↑↑** reduction for SMG (−15.2 kg) vs. placebo (−2.8 kg). At 80-week follow-up (28 weeks post-treatment), reduction remained superior for SMG (−8.7 kg) vs. placebo (−1.2 kg). - % patients attaining BW loss ≥ 5% larger for SMG (86%) vs. 26% after placebo.
		n = 66, and with prediabetes	Placebo Equivalent doses, 52 weeks	
[107] [STEP TEENS]	Placebo-controlled, double-blind trial	n = 119 adolescents with obesity ^‡^	SMG Initial dose of SMG 0.25 mg, with a double dose every 4 weeks up to 2.4 mg/week, 68 weeks	- % patients achieving normal weight or overweight higher for SMG (42.3%) vs. 12.9% placebo. - Improvement of at least one BMI category greater for SMG (73.7%) vs. 19.0% placebo.
		n = 60 adolescents with obesity ^‡^	Placebo Equivalent doses, 68 weeks	

* Comorbidities related to fat mass: hypertension, dyslipidemia, obstructive sleep apnea, or cardiovascular disease; except diabetes. ^†^ Type 2 diabetes, excluding those with HbA1c ≥ 6.5% (48 mmol/mol) or whose diabetes is uncontrolled and unstable with diabetic retinopathy or maculopathy. ^‡^ BMI ≥ 95% percentile or BMI ≥ 85% percentile with at least one weight-related comorbidity (including hypertension, dyslipidemia, obstructive sleep apnea, or type 2 diabetes). BW—body weight, ↑—higher, ↑↑—significantly higher.

### 3.4. Switching to Semaglutide (SWITCH-SEMA)

The Switching to Semaglutide (SWITCH-SEMA) studies investigated the effects of switching from other diabetes treatments, specifically other GLP-1 receptor agonists or diabetes medications, to SMG. The SWITCH-SEMA studies were significant in understanding the benefits of switching to SMG, particularly in terms of the efficacy, safety, and tolerability of SMG compared to the previous medication.

The SWITCH-SEMA 1 study [119] randomized 110 participants with DM2 who were receiving 0.9–1.8 mg/day of liraglutide or 0.75 mg/week of dulaglutide to continue their treatment protocol or switch to subcutaneous SMG (1.0 mg per week). This study showed that switching to SMG improved glycemic control and treatment satisfaction, with a greater reduction in body weight. A sub-analysis of this study including 58 participants suspected of having non-alcoholic fatty liver disease (NAFLD) highlighted that switching to SMG could be beneficial by reducing the fatty liver index [120].

The SWITCH-SEMA 2 study [121] randomized 174 participants with DM2 who were receiving medication with a dipeptidyl peptidase-4 inhibitor (DPP-4i) to either continue their protocol with DPP-4i or switch to oral SMG (initial doses of 3 mg progressing to 14 mg per day). This study showed that although there was a risk of developing gastrointestinal symptoms after starting SMG (seven participants left the study due to gastrointestinal symptoms), switching from DPP-4i to oral SMG had a more significant result on weight loss but could also be beneficial in terms of glycemic control and metabolic abnormalities in people with DM2 associated with high HbA1c levels and insulin resistance. A sub-analysis of this study including 146 participants showed that switching to SMG had a clinically relevant impact on the function of pancreatic beta cells (responsible for producing and releasing insulin), which the authors estimate was presumably through intercellular communication between liver tissue and beta cells [122].

### 3.5. Benefits of Semaglutide on the Cardiovascular System

While it is widely recognized that obesity is a major risk factor for cardiovascular disease [19,123,124], interventions that can achieve effective and lasting weight loss and that concomitantly reduce cardiovascular risk are still limited and challenging to implement consistently. Instead, progress in reducing cardiovascular risk has been achieved through medications indicated for the control of dyslipidemia, hyperglycemia, blood pressure, heart failure, inflammation, and/or thrombosis [125]. Thus, it is important to understand whether SMG may, in addition to weight loss, also have any beneficial impact on the risk of cardiovascular disease or events.

In parallel with the STEP studies, the Semaglutide Effects on Cardiovascular Outcomes in People with Overweight or Obesity (SELECT) studies were also conducted to evaluate cardiovascular benefits in individuals with obesity or overweight. SMG has been shown to promote body weight reduction, improve blood glucose, decrease cardiovascular events in people with diabetes (SUSTAIN and PIONEER studies), and may also result in additional cardioprotective effects [126]. The SELECT studies showed that SMG (Wegovy^®^) in addition to significant weight loss of -10% in the long term (208 weeks) in individuals with obesity or overweight without diabetes and with pre-existing cardiovascular disease [127] also reduces the risk of death from cardiovascular causes, non-fatal myocardial infarction, or non-fatal stroke by 20% [128], which was independent of baseline HbA1c or HbA1c change during the study [129]. The SELECT studies were important in showing that 2.4 mg of subcutaneous SMG (Wegovy^®^) can influence long-term cardiovascular outcomes, strengthening its role in reducing cardiovascular risk beyond weight loss.

The Semaglutide Treatment Effect in People with Heart Failure with Preserved Ejection Fraction (STEP-HFpEF) studies were also conducted in parallel with the STEP studies, but with the aim of evaluating the effect of SMG in a subgroup of individuals with obesity and heart failure with preserved ejection fraction. The “STEP-HFpEF DM” studies include individuals with obesity and heart failure, but also with DM2 [130]. Both groups of studies show a greater reduction in body weight with SMG compared with placebo [131,132]. In addition, these studies showed that 2.4 mg of subcutaneous SMG (Wegovy^®^) improved cardiac remodeling, reduced cardiovascular symptoms and inflammation, and decreased physical limitations [50,51,52,133,134,135]. These studies have allowed to expand the indications for SMG to individuals with obesity (with or without DM2) with heart failure and preserved ejection fraction. The effects on weight loss were greatest in women [136], and the remaining benefits more pronounced in those receiving loop diuretics prior to SMG administration [137].

### 3.6. Other Relevant Studies of Semaglutide

In addition to the cardiovascular system, studies have also been conducted investigating the effect of 1.0 mg of subcutaneous SMG (compared with placebo) on the renal system in individuals with DM2 and chronic kidney disease—the Evaluate Renal Function with Semaglutide Once Weekly (FLOW) studies [138]. The FLOW studies have shown that SMG significantly reduces cardiovascular and renal adverse events, regardless of history of heart failure, initial severity of kidney disease, or concomitant use of Sodium Glucose Cotransporter 2 (SGLT2) inhibitors [139,140,141,142]. The FLOW studies were allowed to end early given the good results obtained [143] and highlighted the potential to expand the indications of SMG for individuals with DM2 and chronic kidney disease for the prevention of clinically relevant kidney events.

In this context, some studies have also been carried out investigating the effect of subcutaneous SMG (compared with placebo) in individuals with non-alcoholic fatty liver disease or non-alcoholic steatohepatitis. SMG has demonstrated benefits in the treatment of patients with non-alcoholic steatohepatitis, including a higher rate of disease resolution compared to placebo, but no significant difference in improvement in fibrosis stage [144]. When combined with firsocostat and/or cilofexor, SMG showed additional benefits, especially in hepatic steatosis and biochemical parameters [145]. In patients with compensated cirrhosis, SMG showed no improvements in fibrosis or resolution of non-alcoholic steatohepatitis [146]. In addition to these results, there were also improvements in health-related quality of life and physical capacity [147]. In patients with non-alcoholic fatty liver disease, although SMG showed no significant difference in liver stiffness, it was able to significantly reduce hepatic steatosis, which, when combined with improvements in liver enzymes and metabolic parameters, suggests a positive impact on liver disease activity and metabolic profile [148]. When comparing SMG 1.0 mg with efinopegdutide 10 mg, SMG showed a smaller reduction in liver fat [149].

The Oral Semaglutide Treatment Effect in People with Obesity (OASIS) study was implemented in individuals with obesity or overweight associated with comorbidities comparing oral SMG (n = 320) versus placebo (n = 307) over 68 weeks [150]. The innovation of this study compared to the PIONEER studies is that they increase the dosage from 3–14 mg daily to a higher daily dose of 50 mg. The dose was progressively increased every four weeks from 3 mg per day to 7 mg, 14 mg, 25 mg, up to a total of 50 mg by the 16th week. Initial results showed that SMG achieved a significantly higher reduction in mean body weight (–15.5 kg and –15.1%) compared to placebo (−2.5 kg and –2.4%). At least a 5% weight reduction was achieved in 85% of those who received SMG, compared with only 26% in the placebo group. These weight loss results with oral SMG 50.0 mg are even superior to those reported at lower doses of oral SMG (PIONEER studies) and similar to subcutaneous SMG 2.4 mg (Wegovy^®^; STEP studies). Oral SMG 50 mg showed a safety profile consistent with previous data on subcutaneous SMG for obesity (STEP) [151] and the GLP-1 receptor agonist class [152]. Thus, oral SMG 50 mg seems to represent an effective option for the treatment of obesity. At the moment, there are more studies from the OASIS group in development that may bring new results in weight loss. OASIS 2 (NCT05132088) compares efficacy and safety of oral SMG 50 mg daily against placebo for 68 weeks, including 198 East Asian adults (including Japan) with obesity or overweight and at least one comorbidity. The OASIS 3 (NCT05890976) study compares the efficacy and safety of oral SMG 50 mg daily against placebo for 44 weeks, including 200 Chinese adults with obesity or overweight and at least one comorbidity. Finally, the OASIS 4 (NCT05564117) study compares the efficacy and safety of oral SMG 25 mg daily against placebo for 64 weeks, including 300 adults with obesity or overweight and at least one comorbidity.

SURPASS studies were conducted to test the effect of another GLP-1 receptor agonist—tirzepatide—in individuals with DM2. Specifically, the SURPASS 2 study [153] compares doses of 5 mg, 10 mg, or 15 mg of subcutaneous tirzepatide against 1 mg of subcutaneous SMG over a 40-week period. This study found a greater loss in body weight with tirzepatide (−7.6 kg for 5 mg, −9.3 kg for 10 mg, and −11.2 kg for 15 mg) compared with SMG (−5.7 kg). The proportion of individuals with weight loss above 5% was also higher for tirzepatide (65–80%) than for SMG (54%). However, it is important to note that the study was carried out by the American company Eli Lilly (which produces tirzepatide), a direct competitor of Novo Nordisk (which produces SMG), which may lead to a risk of study funding bias. In addition, the doses of SMG used are lower (1.0 mg) than those implemented in the most recent studies (2.4 mg), which may affect the results.

A recent systematic review of randomized controlled trials on the use of GLP-1 agonists for weight loss among adults without diabetes showed that, compared to placebo, tirzepatide (15 mg weekly) led to up to 17.8% weight loss after 72 weeks, SMG (2.4 mg weekly) up to 13.9% after 68 weeks, and liraglutide (3.0 mg daily) up to 5.8% after 26 weeks. Retatrutide (12 mg weekly) showed the highest weight loss at 22.1% after 48 weeks. Other GLP-1 agents also demonstrated varying efficacy. Adverse events were common (80–97% vs. 63–100% with placebo), primarily gastrointestinal (nausea, vomiting, diarrhea, constipation). Discontinuation due to adverse effects ranged from 0% to 26% (vs. 0% to 9% with placebo) [154].

### 3.7. Clinical Relevance

The administration of subcutaneous or oral SMG, in addition to its effects on reducing HbA1c concentration and on cardiovascular, renal, and hepatic variables, has a significant effect on body weight reduction. The effect on body composition has been shown to be dose-dependent, with higher doses having a superior effect on weight loss. The effect on body composition was not only absolute weight loss but also a reduction in waist circumference [155,156,157]. To the same extent, subcutaneous (2.4 mg) or oral (50 mg) SMG has an effect on reducing ad libitum energy intake, appetite and satiety, and food cravings, while also improving the control of food intake [100,158,159].

When compared with placebo groups, subcutaneous or oral SMG (regardless of dose) was always significantly superior in reducing body weight. Similarly, SMG was generally significantly superior in weight loss when compared with other glucose-controlling drugs (e.g., sitagliptin, insulin glargine or aspartic, canagliflozin or empagliflozin) or other GLP-1 receptor agonists (e.g., exenatide, liraglutide or dulaglutide). When transitioning from medication from other GLP-1 receptor agonists to SMG, it is still possible to achieve additional significant weight loss with SMG [119]. The same results of SMG are demonstrated in systematic reviews with meta-analyses [98,155,156,157,160], which encompass most of the studies described above in the tables. However, it must be considered that weight loss may be temporary since, after cessation of SMG, at the end of one year, a substantial percentage of the lost weight is regained [93,99].

SMG is originally and still commonly administered subcutaneously. Even so, the good initial results with the oral formulation (Rybelsus^®^), although with a lower weight loss compared to subcutaneous administration, encourage that the oral option may gain popularity due to its convenience and ease of administration, which enhance logistical simplicity and integration into the routine and may lead to greater adherence to treatment. Following the good results of the PIONEER studies investigating the Rybelsus^®^ formulation, the OASIS 1 study [150] found superior weight loss with higher doses of oral SMG (50 mg) in individuals with obesity or overweight associated with comorbidities. These results may lead to greater adherence to the oral formulation of SMG and thus increase the popularity of this drug as long as there is no significant increase in adverse effects. At this time, the OASIS 1 study showed that oral SMG 50 mg had a safety profile consistent with those previously reported for subcutaneous SMG in obesity and with other drugs in the GLP-1 receptor agonist class. The OASIS 2–4 studies should bring news in the near future on the potential of oral SMG (50 mg).

The use of SMG is essentially indicated for the control of DM2 (Ozempic^®^) and obesity or overweight associated with comorbidities (Wegovy^®^). Following the good results in the cardiovascular, renal, and hepatic systems, it is likely that the indications of SMG will be expanded to the treatment or control of other pathologies that may benefit from the reduction in body weight. The good results in reducing BMI in adolescents demonstrated in the STEP TEENS study [107] may also enhance the expansion of SMG indications for adolescents with obesity and thus help combat childhood obesity.

As with any medication, one must consider the potential adverse effects. The most common adverse effects of SMG are nausea, vomiting, diarrhea, cholelithiasis, and constipation [155], which are typical of this class of drugs [161]. However, it should be considered that these adverse effects are considered mild to moderate, and there is no significant probability of serious adverse effects [155] or an adverse reaction to SMG when compared to placebo [157]. Although SMG and several other therapies have been associated with an increased risk of adverse events, SMG has demonstrated substantially greater weight loss benefits than other therapies with a similar risk of adverse effects [42]. Still, it is important to note that the percentage of participants discontinuing SMG due to adverse effects and gastrointestinal side effects is statistically significant when compared to placebo [155].

Oral or subcutaneous SMG has been shown to be a cost-effective long-term therapy compared with other non-surgical weight loss strategies or other GLP-1 receptor agonist drugs [162,163]. Subcutaneous SMG (2.4 mg) is cost-effective in reducing weight in individuals with obesity or overweight associated with comorbidities when compared with a group with no treatment, a group with only diet and exercise, and all other groups with other anti-obesity drugs (liraglutide 3 mg, phentermine-topiramate, and naltrexone-bupropion) [162]. Oral SMG (14 mg) has demonstrated health benefits similar to those of subcutaneous SMG (1.0 mg) and superior to those of dulaglutide (1.5 mg) and liraglutide (1.8 mg) in individuals with DM2 inadequately controlled with oral antidiabetics. In addition, oral SMG was less expensive than the subcutaneous formulation and the other GLP-1 receptor agonist drugs (dulaglutide and liraglutide), making it a more cost-effective option [163].

The cost of SMG treatment is an important factor that should be carefully considered, as it may influence its widespread adoption into clinical practice. The cost varies significantly across countries, which can affect accessibility. In the United States, the monthly list price for Ozempic^®^ and Rybelsus^®^ is approximately $936, while Wegovy^®^, a higher-dose version for weight management, is priced at $1349 per month. In contrast, prices in other countries are much lower: for example, Ozempic^®^ costs $83 per month in France, $93 in the United Kingdom, $96 in Sweden, $87 in Australia, and $147 in Canada [164]. High demand has also led to supply shortages, affecting new prescriptions. In countries such as the Czech Republic, Romania, Serbia, and Spain, both injectable and oral forms of SMG are available, providing patients with more treatment options. However, in other regions, only the injectable form is available [165].

Given the evidence demonstrating that SMG is a cost-effective approach in the treatment of DM2 and obesity, it is crucial that all stakeholders and policy-makers consider its inclusion, both in oral and subcutaneous formulations, in treatment protocols for these conditions. This inclusion should be accompanied by state support for financing the drug. In addition, the affordability and availability of SMG in the public health system should be evaluated, promoting a positive impact on the high use of society’s financial resources and sustainability of health systems in the treatment of DM2 and obesity [166,167,168,169].

A real-world retrospective cohort study investigated factors influencing weight loss response to subcutaneous GLP-1 analogs (86% of patients were prescribed subcutaneous SMG, and 14% were prescribed liraglutide) in 483 adults with obesity (BMI ≥30 kg/m²) at a multidisciplinary clinic in Vancouver, Canada (2018–2021). Over an average follow-up of 17.3 months, participants experienced a mean total body weight loss (%TBWL) of 12.2%. Response categories included non-response (<5% TBWL, 17.8%), moderate response (5–15% TBWL, 48.4%), and hyper-response (>15% TBWL, 33.8%). Multivariable analysis identified female sex as a predictor of hyper-response, while age, diabetes status, baseline BMI, sedentary behavior, anxiety, and depression showed no significant associations. Findings suggest that sex may influence weight loss outcomes with GLP-1 analogs, warranting further research to identify additional predictive biomarkers [170].

### 3.8. Limitations of the Review and Literature

This work presents a narrative review of the literature, using the database search methodology used in systematic reviews for a more systematic identification of relevant studies. Narrative reviews are inherently limited in relation to systematic reviews, having a lower rigor in the selection and inclusion of all available studies and in the quantitative synthesis analyses that can be performed [171]. Still, the objective of this review was to summarize the results for weight loss from the main studies investigating SMG. An initial search was carried out in PubMed, and several complementary searches were carried out to identify studies that were omitted in the initial search in PubMed. Although a large selection of relevant studies has been included, there may be other studies of SMG that have not been included. However, the results presented in this work are in the same line as those reported in systematic reviews with meta-analyses [155,156,157].

The included trials show a low risk of selection bias due to the random allocation of participants to the different groups [172]. To the same extent, most studies demonstrated a low risk of detection bias, as a double-blind outcome evaluation system was often implemented [172] in addition to, where applicable, a double dummy system. In addition, most studies included large numbers of participants, which helps to increase the certainty of the evidence due to the low risk of imprecision [173]. Despite these positive points, it must be considered that many studies have demonstrated a high risk of attrition bias [172] due to a significant proportion of participants discontinuing the use of SMG due to gastrointestinal adverse effects. Despite a potentially low risk of selective reporting bias [172] since the studies had registered the study protocol in the ClinicalTrials.gov database, there may always be, although potentially of low magnitude, a risk of funding bias (the studies were funded by the company Novo Nordisk, which markets SMG) that may potentiate a risk of selective reporting bias or publication bias due to financing [174,175,176,177]. In fact, the studies reported that the study design and protocol had been designed by Novo Nordisk, but with the caveat that the study implementation and analysis of the results had been performed by independent researchers to mitigate the risk of funding bias.

Although there are several studies comparing SMG with other GLP-1 receptor agonist drugs, the literature in this context is still scarce. The studies presented in this work that show superiority of SMG over other GLP-1 receptor agonists are funded by Novo Nordisk, and the studies [153,178] that show tirzepatide’s superiority over SMG are funded by Eli Lilly, Novo Nordisk’s American rival in the formulation of GLP-1 receptor agonist drugs. In this sense, these studies are not free from high risk of funding bias, and it is therefore essential to understand whether these results are replicable by independent groups of researchers who are not funded by the pharmaceutical industry.

Another limitation of the literature is inherent to the limited time in which SMG is available on the market. Despite some studies that have been published with 4 years of follow-up, more time will be needed to better understand whether the continued use of SMG may lead to long-term adverse effects. For example, weight loss with the use of GLP-1 receptor agonist medication seems to be associated with a significant loss of lean mass as well [179], which may later lead to physical and functional disability or even sarcopenia [180]. There are currently ongoing studies exploring strategies to mitigate muscle loss with the medication of GLP-1 receptor agonists, such as bimagrumab (NCT05616013) and enobosarm (NCT06282458), which may offer solutions to preserve muscle mass in individuals undergoing weight loss treatments [180]. In addition, in long-term studies, it will be important to understand the potential retention effect of weight loss since the few studies carried out in this context show that much of the weight lost is regained [93,99].

## 4. Future Directions

In the context of constant innovation in this area, some recent studies have been published that try to innovate GLP-1 receptor agonist drugs. A recent randomized, double-blind study [153] evaluated the efficacy and safety of combining SMG with the amylin analog cagrilintide (CagriSema) in participants with DM2. Participants were randomized to receive CagriSema, SMG (2.4 mg), or cagrilintide (2.4 mg) once weekly for 32 weeks. The CagriSema combination formulation achieved greater weight loss (–15.6%) compared to SMG (–5.1%) and cagrilintide (–8.1%). Another recent randomized double-blind study [181] compared a new formulation of dual GLP-1 receptor agonist and glucagon—survodutide—against 1.0 mg of subcutaneous SMG (n = 59) and against placebo (n = 50) in individuals with DM2. The dose of survodutide was 0.3 mg, 0.9 mg, 1.8 mg, or 2.7 mg once weekly, or 1.2 mg or 1.8 mg twice weekly, comprising a total of 302 participants, ranging from 49 to 52 per group. The study ran for only 16 weeks. The reduction in body weight with survodutide was dose-dependent and comparable with SMG, with only the dose of 1.8 mg twice weekly showing a superior effect when compared to SMG.

These two previous studies show a cycle of constant innovation in the development of GLP-1 receptor agonists that may bring innovations in the treatment of obesity and other pathological conditions. In fact, in a search carried out on the ClinicalTrials.gov website (20 November 2024), a total of 458 registered studies for the use of SMG were found, many of them still active, either recruiting participants or that had not yet started the recruitment phase. This research highlights that there are several studies under development that may bring additional results in the continuation or replication of the use of SMG in patients with obesity and/or DM2 but also investigate new combinations (e.g., combination with dehydrated cannabidiol—NCT06648031) compared to new drugs (NCT06649344, NCT06604624, NCT06579105, NCT06577090, NCT06497049, NCT06282458, and NCT05616013) and may even expand the indications for other pathological conditions (associated or not with obesity or DM2), such as steroid-induced diabetes (NCT06318442), acute myocardial infarction (NCT06557811), autosomal dominant polycystic kidney disease (NCT06582875), steatohepatitis associated with metabolic dysfunction (NCT06492330 and NCT06374875), obesity-related asthma (NCT05254314), cystic fibrosis (NCT05788965), advanced interstitial lung disease (NCT05746039), psoriasis vulgaris (NCT06475586), systemic scleroderma (NCT06149260), idiopathic intracranial hypertension (NCT06361823 and NCT06027567), post-acute stroke (NCT05630586 and NCT05920889), neuroleptic-related prediabetes in individuals with schizophrenia (NCT05193578), Alzheimer’s (NCT06072963 and NCT05891496), functional hypogonadism (NCT06489457), polycystic ovary syndrome (NCT05646199, NCT05702905, NCT06222437, and NCT05819853), endometrial hyperplasia (NCT05829460), Klinefelter’s syndrome (NCT05586802), or even in alcohol consumption disorder (NCT05520775, NCT05891587, NCT05895643, and NCT05892432), opioid use (NCT06548490), or cocaine use with and without human immunodeficiency virus (NCT06691243), among many other potential therapeutic indications.

Despite the constant innovative cycle of measurement of GLP-1 receptor agonists, and specifically SMG, there are still some issues and uncertainties that should be explored in future studies:

(1) Considering that obesity is a chronic, progressive and relapsing disease [182], it is still necessary to determine the optimal duration of SMG therapy for weight control and to identify potential needs for medication adjustments based on individual characteristics. Future studies are needed to investigate the durability of weight loss retention and metabolic benefits, especially based on the less encouraging results of regaining lost weight [93,99], and it is not yet clear whether the trajectory of weight regain continues in subsequent years. Weight regain was faster and more significant in individuals who lost more weight and when lifestyle modification intervention was removed [99]. It is important to develop strategies to prevent or mitigate this phenomenon of weight regain, which may include a gradual reduction in the dose of SMG, participation in specific lifestyle programs after discontinuation of SMG, or the determination of criteria for new SMG treatment cycles in case of rapid weight regain [183].
(2) SMG-induced gastrointestinal adverse effects, although common, are transient and mild to moderate in severity. The safety profile of SMG compared to other pharmacological therapies is important, as many other therapies have an adverse effect profile that contributes to its poor adherence [161,184]. This finding will be important given the discontinuation of SMG due to gastrointestinal adverse effects. In the context of continuing to take SMG, it is important to understand the risk of more serious long-term adverse effects, such as pancreatitis, intestinal obstruction, and gastroparesis [185], or even the effect of loss of lean mass [179].
(3) It is also important to investigate the potential effects of SMG on hormonal contraception, pregnancy, or breastfeeding [186]. Animal studies exposed to GLP-1 receptor agonists during pregnancy have shown adverse outcomes such as decreased fetal growth and abnormalities, but human studies have not shown a significant risk of developing birth defects [187,188,189,190]. Although periconceptional exposure to GLP-1 receptor agonists has not shown an increased risk of malformations, data on complications such as fetal growth restriction or embryonic death are lacking [188].
(4) Given the variability in weight loss with SMG, it will be important to find prognostic predictors that identify those who do not respond to SMG (loss of less than 5% of weight) and those who are super-responders (loss of more than 20% of weight) [183]. At this time, two factors have already been identified, being the coexistence of DM2 [95,101] and male gender [170,191], that appear to decrease the effect of SMG on weight loss (with contradictory results regarding diabetes [170]). In addition, age may be a factor in discontinuing SMG intervention, as it is higher in elderly individuals [70]. It will be essential that future studies are able to explore other potential predictors such as demographic characteristics (gender, ethnic origin, age), metabolic parameters (baseline BMI, HbA1c, fasting blood glucose, markers of insulin resistance, lipid profile), eating behaviors, and genotype [183]. The study of epigenetics for the identification of specific genotypes and phenotypes [192,193] could help determine algorithms and predictive models for personalized decision-making to optimize therapeutic benefit and minimize associated risks [193,194].
(5) Bariatric surgery is a therapeutic option for individuals with obesity [195], reducing long-term all-cause mortality and the incidence of obesity-related diseases [196,197]. However, weight regain after bariatric surgery is common [198]. Retrospective studies prove the efficacy and safety of SMG in maintaining weight loss after bariatric surgery, with an average body weight reduction of 9.8–10.3% after 6 months [199,200,201]. Future studies should investigate whether SMG could play an important role as a pre-bariatric surgery intervention or even when combined with endoscopic bariatric therapies such as intragastric balloons or endoscopic sleeve gastrectomy [202]. At the moment, efforts are being made to investigate the role of SMG after bariatric surgery through the BARI-STEP study (NCT05073835), which aims to determine the role of SMG in individuals who have experienced insufficient weight loss or excessive weight gain after bariatric surgery.

## 5. Conclusions

SMG, in its subcutaneous (Ozempic^®^) and oral (Rybelsus^®^) formulations, has been shown to be significantly effective in the treatment of DM2. In addition to improving glycemic control, SMG promotes significant reductions in body weight, with additional benefits on cardiovascular health and the prevention of metabolic complications. Promising results in weight loss with SMG in individuals with DM2 have led to the expansion of indications for the treatment of obesity or overweight associated with comorbidities (Wegovy^®^). Clinical studies confirm the superiority of SMG over most other treatments in the same class of GLP-1 receptor agonists, highlighting its ability to induce weight loss safely and effectively, even in populations with comorbidities.

Oral SMG showed less substantial initial results in weight loss, but the new formulations with doses of 50 mg demonstrated a significant weight loss comparable to the subcutaneous formulation. Trials (OASIS) are underway to further evaluate the 50 mg dose of oral SMG, which may become an attractive option for more widespread use if it shows similar weight loss results to the subcutaneous formulation without a significant increase in adverse events.

Future directions for the use of SMG involve expanding its indications beyond the treatment of DM2 and obesity by exploring new therapeutic applications and novel clinical strategies. Prospective studies should focus on assessing SMG’s potential for managing other conditions that could benefit from weight loss, as well as investigating the combination or sequential use of SMG with emerging approaches. A critical area for future research is understanding the long-term effects of continued SMG use, evaluating its effectiveness, and determining how long weight loss can be maintained. Identifying predictive factors for responders and non-responders to SMG could enable the development of personalized treatment algorithms.

## Figures and Tables

**Table 1:** Results of the initial randomized, placebo-controlled, double-blind trial studies (phase 2 and phase 3a) of semaglutide (SMG) in patients with type 2 diabetes.

Reference	Population	Intervention	Results in Body Weight
[59]	n = 270	SMG 0.1 to 0.8 mg/week or progressive dose up 1.2 to 1.6 mg/week (sc, 12 weeks)	- ↑ reduction for SMG 0.8–1.6 mg (−3.4 to −4.8 kg) vs. placebo (−1.2 kg). - ↑ reduction for SMG 0.8 and 1.6 mg vs. liraglutide 1.8 mg (−2.6 kg) and for SMG 0.8 mg, and 1.6 mg vs. liraglutide 1.2 mg (−1.9 kg). - % patients with BW ≥ 5% increased in a dose-dependent manner (2%, 7%, 13%, 38%, 51% and 64% with SMG 0.1–1.6 mg) vs. 18% and 14% of those with liraglutide 1.2–1.8 mg, and vs. 13% after placebo.
	n = 46	Placebo Weekly dose (sc, 12 weeks)	
	n = 95	Liraglutide Progressive dose up 1.2 to 1.8 mg/day (sc, 12 weeks)	
[60]	n = 69	SMG 1.0 mg/week (sc) or	- ↑ reduction with oral (−2.1 kg to −6.9 kg, according to dosage) or sc SMG (−6.4 kg) vs. placebo (−1.2 kg). - ↑↑ reduction for oral SMG dosages of 10 mg or > vs. placebo (−0.9 to −5.7 kg, according to dosage).
	n = 350	2.5 to 40 mg/week with or without progressive oral dosing (26 weeks)	
	n = 71	Placebo Weekly dose (oral, 26 weeks)	

Abbreviations: BW—body weight, sc—subcutaneous, ↑—higher, ↑↑—significantly higher.

## Data Availability

Data are contained within the article.

## Abbreviations

The following abbreviations are used in this manuscript:

BMI	Body Mass Index
DM2	Type 2 Diabetes
DPP-4	Dipeptidyl peptidase-4
DPP-4i	Dipeptidyl peptidase-4 inhibitor
EMA	European Medicines Agency
FDA	Food and Drug Administration
GLP-1	Glucagon like peptide-1
HbA1c	Glycated haemoglobin
NAFLD	Non-alcoholic fatty liver disease
SGLT2	Sodium Glucose Cotransporter 2
SMG	Semaglutide
WHO	World Health Organization
